# 3D Model of Carbon Diffusion during Diffusional Phase Transformations

**DOI:** 10.3390/ma17030674

**Published:** 2024-01-30

**Authors:** Łukasz Łach, Dmytro Svyetlichnyy

**Affiliations:** AGH University of Krakow, Faculty of Metals Engineering and Industrial Computer Science, al. Mickiewicza 30, 30-059 Krakow, Poland; svetlich@metal.agh.edu.pl

**Keywords:** phase transformation, carbon diffusion, frontal cellular automata, lattice Boltzmann method, CUDA

## Abstract

The microstructure plays a crucial role in determining the properties of metallic materials, in terms of both their strength and functionality in various conditions. In the context of the formation of microstructure, phase transformations that occur in materials are highly significant. These are processes during which the structure of a material undergoes changes, most commonly as a result of variations in temperature, pressure, or chemical composition. The study of phase transformations is a broad and rapidly evolving research area that encompasses both experimental investigations and modeling studies. A foundational understanding of carbon diffusion and phase transformations in materials science is essential for comprehending the behavior of materials under different conditions. This understanding forms the basis for the development and optimization of materials with desired properties. The aim of this paper is to create a three-dimensional model for carbon diffusion in the context of modeling diffusional phase transformations occurring in carbon steels. The proposed model relies on the utilization of the LBM (Lattice Boltzmann Method) and CUDA architecture. The resultant carbon diffusion model is intricately linked with a microstructure evolution model grounded in FCA (Frontal Cellular Automata). This manuscript provides a concise overview of the LBM and the FCA method. It outlines the structure of the developed three-dimensional model for carbon diffusion, details its correlation with the microstructure evolution model, and presents the developed algorithm for simulating carbon diffusion. Demonstrative examples of simulation results, illustrating the growth of the emerging phase and affected by various model parameters within particular planes of the 3D calculation domain, are also presented.

## 1. Introduction

The microstructure plays a crucial role [1] in forming the mechanical properties of materials. Materials engineers strive to manipulate the microstructure [2] through processes such as heat treatment, mechanical processing, and other methods to achieve the desired material characteristics. In cases of metals, ceramics, polymers, and other materials, the microstructure significantly influences their strength [3], hardness, plasticity, brittleness, and many other mechanical properties. One of the fundamental phenomena that affects the development of the microstructure in materials is phase transformation [4].

Phase transformations in materials depend on a material’s chemical composition and refer to changes in the state of matter or internal structure of a material in response [5] to changes in conditions, such as temperature and pressure.

Phase transformations in steels can be divided into two main groups [6]: diffusional transformations [7] and diffusionless transformations [8]. Diffusionless transformations are usually much faster [9] than diffusional transformations, and they occur under extreme cooling (and heating) or deformation conditions [10]. On the other hand, diffusional transformations require longer times (especially at low temperatures [7]), allowing for the diffusion of atoms and a more gradual change in the crystalline structure.

Among recent works related to diffusional phase transformations, the work of Wang et al. [11] and Liu et al. [12] are worthy of attention. Wang et al. present progress in research on the diffusional phase transformations of Fe–C alloys under high magnetic fields. The authors show that a high magnetic field can provide the driving force for transformations and promote ferrite and pearlite transformations and carbide precipitation and that determining the laws that govern the changes in physical parameters, such as the interfacial energy and diffusion coefficients under a high magnetic field, should be the basis of future research on diffusional phase transformations. Liu et al. published a review on the modeling and simulations of solid-state diffusional phase transformations in metals and alloys. In the revision, the authors noted that the traditional diffusion-controlled and interface-controlled models fail to correctly describe the growth kinetics of the new phase over the entire course of the transformation, and the empirical and analytical models only provide average information on the macroscale.

The studies of carbon diffusion during phase transformations retain their significant importance [13] in materials science, engineering, and various industrial applications. Understanding how carbon diffuses during phase transformations is crucial to designing and optimizing materials with specific properties. For example, in the development of advanced steels, knowledge of carbon diffusion during phase changes helps engineers tailor [14] the strength, hardness, and corrosion resistance of the material. Carbon diffusion during phase transformations also provides insight into the thermodynamics and kinetics of these processes [12]. Researchers can study the energy barriers [15] involved and the time required for transformations [16], thereby leading to better control and predictability. In industries such as steel manufacturing, the study of carbon diffusion is vital for optimizing processes and reducing energy consumption. It allows for the development of more energy-efficient methods while maintaining product quality.

Advanced computational methods [12] are very helpful in understanding carbon diffusion during diffusional phase transformations, which is a highly complex and multifaceted process. Advanced computational methods provide [17] the ability to model and simulate intricate interactions, which offer insights that are difficult to obtain by analytical or experimental means alone. Computational methods allow researchers to obtain quantitative results on the diffusion of carbon atoms, such as concentration profiles [18], diffusion coefficients [19], and diffusion rates [20]. These insights are essential for making accurate predictions and optimizing material properties. Topics related to carbon diffusion in steels are important when taking into account different model studies. Lee et al., for example, proposed a simple and computationally efficient equation for carbon diffusion in austenite in iron–carbon alloys, as well as in steels alloyed with a variety of elements [21]. The authors of the work presented different equations for the diffusivity of carbon in austenite and developed empirical equations to predict carbon diffusivity in austenite as a function of chemical composition and temperature for the iron–carbon-based multicomponent alloy steels.

Various phenomena and processes in engineering can be modeled using different numerical methods. The choice of a specific method depends on the nature of the problem, the availability of data, the precision of the expected result, and the computational resources [22,23]. Often, various numerical methods are combined to achieve a more comprehensive analysis of a given phenomenon [24]. Different methods can also be employed to model phase transformations.

Recently, Yamanaka [19] presented recent advances in phase-field modeling and simulation of solid-state phase transformations in iron and steel, with particular attention paid to the modeling of austenite-to-ferrite, pearlitic, bainitic, and martensitic transformations. The author emphasized that the phase-field method can be applied to various solid-state phase transformations in iron and steel because it can simulate multi-physics phenomena, such as the migration of interfaces, the diffusion of solute atoms, and the evolution of the stress–strain fields during phase transformations. The author also noted that the current phase-field models are generally unable to easily provide quantitative simulation results for the solid-state phase transformations taking place in multicomponent steels that contain substitutional alloying elements. Lahiri presented a review on phase-field modeling of phase transformations in multicomponent alloys [25], where he noticed that, in view of the versatility and quantitative accuracy of the discussed PF models, it can be anticipated that simulations of microstructure evolution using such quantitative PF models will continue to accelerate the design and development of novel multicomponent alloys in the future. Lv et al. [26] found that numerical uncertainty and parameter sensitivity have a major impact on the simulation results in all kinds of phase-field models.

FCA and the LBM are highly effective approaches for simulating various phenomena and processes in the field of materials science. Recently, Svyetlichnyy analyzed the computational time complexity of Frontal Cellular Automata (FCAs) and compared them with Classic Cellular Automata (CCAs) [27], including phase transformation [28]. FCA has a very wide range of applications in the context of modeling various processes and phenomena occurring in materials. This method, among others, has been used successfully for the modeling of recrystallization [29], additive manufacturing [29,30], and rolling [31]. Recently, parallel computing modeling is also being realized on a larger scale; for example, Zhu et al. [32] realized the three-dimensional multi-phase-field simulation of a eutectoid alloy based on an OpenCL parallel.

This paper presents a 3D model that focuses on the diffusion of carbon in diffusion phase transformations. This model is one of the most important components of a sophisticated system designed to model such transformations. Built upon two modeling methods, FCA and the LBM, the 3D model incorporates the utilization of hybrid computing systems (GPU and CPU) for calculations. Fundamental details regarding the FCA method and the LBM are presented. The structure and primary assumptions of the model are also demonstrated. This paper presents some modeling results of the transformation obtained by this model. Parallel computing is performed on NVIDIA graphics cards with CUDA architecture and with visualization using Open GL.

## 2. Lattice Boltzmann Method

The LBM originated from a variant of the cellular automata method known as the lattice gas automaton (LGA) [33]. The basis of the method is to solve the Boltzmann transport equation:

(1) $$\frac{\partial f}{\partial t}+\frac{\partial f}{\partial x}\cdot \xi +\frac{F}{m}\cdot \frac{\partial f}{\partial e}=\Omega$$

where *f*(***x***,***u***,*t*) represents the particle distribution function, ***x*** and ***ξ*** denote phase space variables (coordinate and velocity), *t* signifies time, ***F*** stands for external force (e.g., gravity), *m* is the mass, ***u*** represents macroscopic velocity, Ω is the collision operator, and ***e*** represents lattice velocities.

Particles are distributed according to velocities and directions, as follows:

(2) $$f=\frac{\rho }{{\left(2\pi RT\right)}^{D/2}}\exp\left(-\frac{{\left(\xi -u\right)}^{2}}{2RT}\right)$$

where *R* is the gas constant, *D* represents the space dimension, *ρ* signifies gas density, and *T* denotes temperature.

Along with the discretization of space and time, the LBM uses the discretization of velocities by dividing them into a limited number of directions and setting the velocities according to the lattice space. The velocities ***ξ*** are discretized to a set of such discrete velocities ***e***. Equation (1) is approximated for characteristic velocities ***e*** determined by the grid and by directions using trapezoidal integration for spatial variables and an explicit Euler scheme for time derivatives. The result of such a discrete approximation is the lattice Boltzmann equation (LBE), which presents an evolution of components of the distribution function as follows:

(3) $$f_{i}\left(x+e_{i},t+1\right)=f_{i}\left(x,t\right)+\Omega_{i}$$

Equation (3) is the foundation of the LBM.

Discretization of two- or three-dimensional space consists of imposing a regular orthogonal grid on a uniform lattice of dimensionless length (the distance between neighboring nodes) equal to one dx = dy = dz = 1. The parameter dt related to the dimensionless time step is also set to unity dt = 1. A fundamental aspect of the system involves selecting a set of velocities ***e***, known as the velocity model, for example, the D2Q9 model, where D indicates dimensionality (D2—two-dimensional model) and Q indicates the number of velocities. In one-dimensional space, the primary models used are D1Q2 or D1Q3, while in two-dimensional space, models such as D2Q4, D2Q5, or D2Q9 are commonly employed. For three-dimensional problems, models like D3Q6, D3Q7, D3Q15, D3Q19, and others find application.

The important element of this equation (Equation (3)) is the collision operator Ω*_i_*. In general, this operator is a complex nonlinear integral. An approximation of this integral can be fulfilled by linearization near a local equilibrium point. Several approximations are developed: single-time, two-time, and multi-time relaxation schemes and a cascade scheme. The first and simplest single-time relaxation scheme was proposed by Bhatnagar, Gross, and Krook and was called the BGK collision operator:

(4) $$\Omega_{i}\left(f\right)=-\frac{1}{\tau }\left[f_{i}\left(x,t\right)-f_{i}^{eq}\left(x,t\right)\right]$$

where *τ*—relaxation time and *f^eq^*—equilibrium distribution function.

The BGK collision operator (Equation (4)) contains an equilibrium distribution function *f^eq^*, which can take the following form:

(5) $$f_{i}^{eq}\left(x,t\right)=w_{i}\rho \left(1+\frac{u\cdot e_{i}}{c_{s}^{2}}+\frac{{\left(u\cdot e_{i}\right)}^{2}}{2c_{s}^{4}}-\frac{u\cdot u}{2c_{s}^{2}}\right)$$

where *ρ*—density, ***u***—lattice velocity, *c_s_*—sound speed in the modeled fluid (for D2Q9), and *c_s_* = $1/\sqrt{3}$, *w_i_*—directional weights (for example, for D2Q9: *w*_0_ = 4/9; *w*_1_ = *w*_2_ = *w*_3_ = *w*_4_ = 1/9; *w*_5_ = *w*_6_ = *w*_7_ = *w*_8_ = 1/36).

The macroscopic variables, density *ρ* and velocity ***u*** (through the momentum *ρ**u***) (Equation (5)), are calculated as follows:

(6) $$\rho =\sum_{i=0}^{b}f_{i}$$

(7) $$\rho u=\sum_{i=0}^{b}f_{i}e_{i}$$

The LBE (Equation (3)) is solved in two stages: collision and streaming. The post-collision form of the initial distribution function is then as follows:

(8) $$f_{i}^{out}\left(x,t\right)=f_{i}^{in}\left(x,t\right)-\frac{1}{\tau }\left[f_{i}^{in}\left(x,t\right)-f_{i}^{eq}\left(x,t\right)\right]+F_{i}$$

The streaming is a direct transfer of components of distribution functions to appropriate neighboring nodes:

(9) $$f_{i}^{in}\left(x+e_{i},t+1\right)=f_{i}^{out}\left(x,t\right)$$

Boundary conditions can be considered as a separate operation or in conjunction with a streaming.

**Figure 1:**
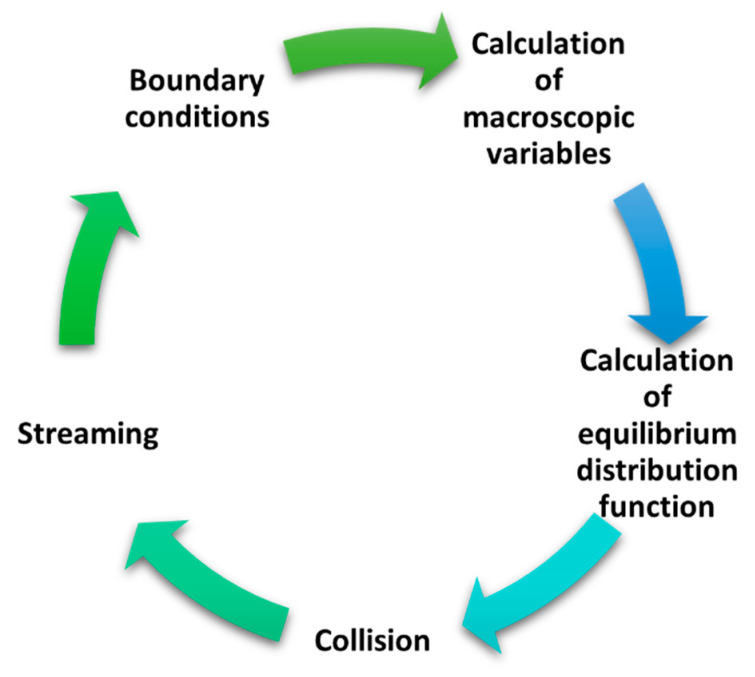
Graphical representation of main algorithm of LBM [33].

Figure 1 shows the general modeling algorithm using the LBM.

A detailed description of the LBM can be found elsewhere [33].

## 3. Frontal Cellular Automata

The frontal cellular automaton is a specific type of computational algorithm based on cellular automaton models. The name “frontal” comes from one of its most widespread applications, namely, grain growth during the microstructure evolution, where the boundary of a growing grain is considered as the front of changes occurring in the structure. The main differences in FCA from classical CA are the reversal of information sending (the cell sends information to its neighbors instead of receiving information from the neighborhood), the introduction of an additional state into the automaton, and the organization of the cells into lists. As a result, only small part of all cells is used in calculations in one time step, every cell is maintained only once in the entire simulation, the time complexity is reduced, and simulation time decreases significantly [27].

**Figure 2:**
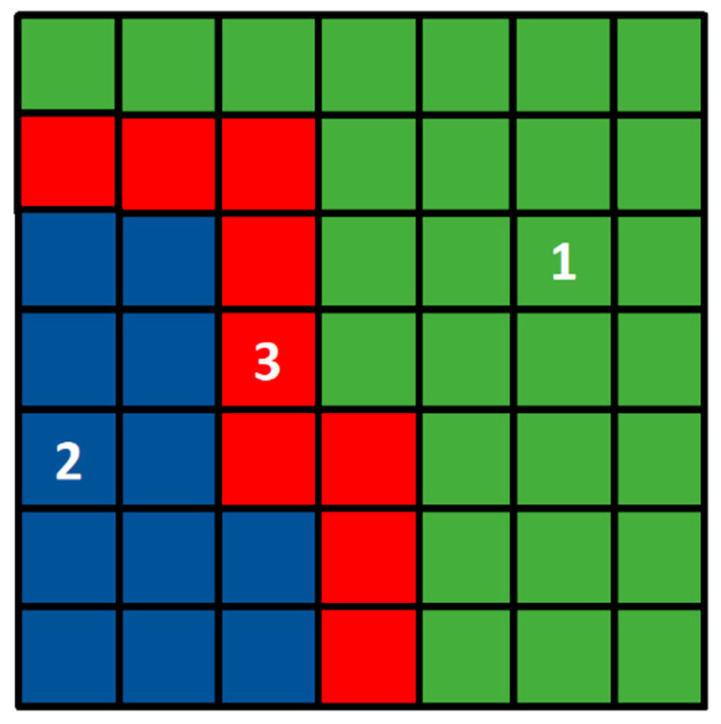
FCA zones: 1—cells in the initial state, 2—cells in the final state, 3—cells participating in computations.

Three areas can be separated during grain growth in the phase transformation process (Figure 2). Transformation does not begin in the first area, and cells remain in the initial state. The transformation ends in the second area: the cells are in the final state, and this state will not change. The third area is between the first two: it separates them, and the transformation occurs in this area. Only the third area is utilized in FCA calculations. Both the first and second zones should be excluded from the calculations in the current step because changes in the cell states within these zones are not expected and cannot occur.

The use of FCA (Frontal Cellular Automaton) instead of conventional cellular automata reduces simulation time not only in 2D models but also in 3D CA, where it is more noticeable. This is because significant spatial areas are excluded from the calculations at each time step, and only a thin layer participates in the computations. In classical CA, each step requires practically the same computational effort, and the time taken for its determination remains unchanged throughout the entire simulation. In contrast, the duration of one time step calculations in FCA depends on the number of participating cells, and it exhibits significant variability, but it always remains a small fraction compared to the duration in classical CA. Each cell in FCA participates in calculations only once, in one time step, during the entire simulation, whereas in classical CA the cells participate in calculations in all time steps.

Details about FCA can be found elsewhere [27].

## 4. 3D Carbon Diffusion Model

**Figure 3:**
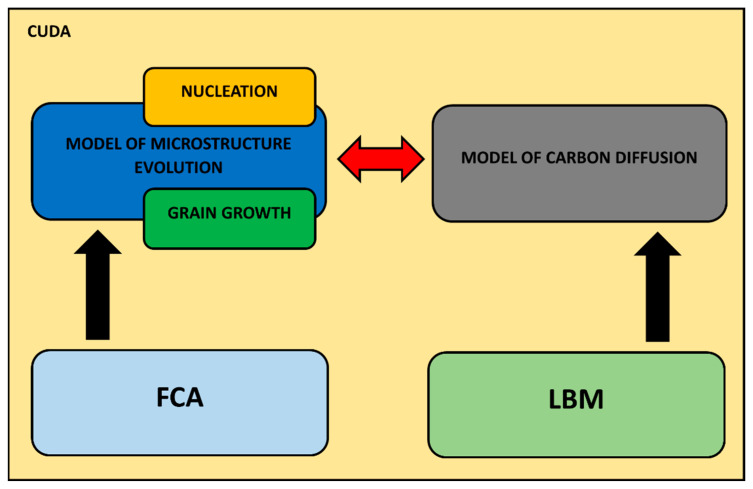
A 3D model of carbon diffusion (based on LBM) and its integration with the microstructure evolution model (based on FCA) (red arrows symbolize the interaction between these models).

A multiscale model, which contains the 3D carbon diffusion model based on the LBM and a microstructure evolution model based on FCA, is shown in Figure 3. This multiscale model was adapted for parallel calculations on GPU with the CUDA architecture.

**Figure 4:**
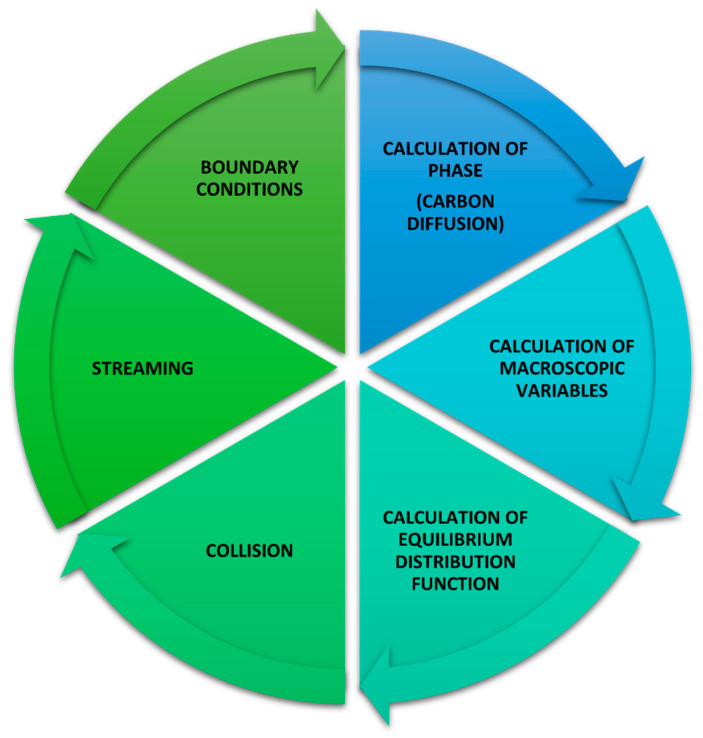
Carbon diffusion in the calculation of phase in the LBM algorithm. Particular colors represent various stages of the cyclically realized algorithm.

Phase separation, carbon diffusion in different phases, and calculation of the phase fraction in cells on the phase’s boundaries are the additional elements implemented into the standard LBM modeling scheme. These elements are shown in Figure 4 as a common block named ‘calculation of phase (carbon diffusion)’.

The diffusion model is based on the 3D Fourier equation [34]:

(10) $$\frac{\partial c}{\partial t}=D\left(\frac{\partial^{2}c}{\partial x^{2}}+\frac{\partial^{2}c}{\partial y^{2}}+\frac{\partial^{2}c}{\partial z^{2}}\right)$$

where *c*—carbon concentration and *D*—carbon diffusion coefficient.

The Bhatnagar–Gross–Krook (BGK) collision operation used in the LBM [33] (Equation (4)) is applied for the calculations.

The equilibrium distribution function is calculated as follows:

(11) $$f_{k}^{eq}=w_{k}c\left(x,y,z,t\right)$$

where *c* is the carbon concentration.

**Figure 5:**
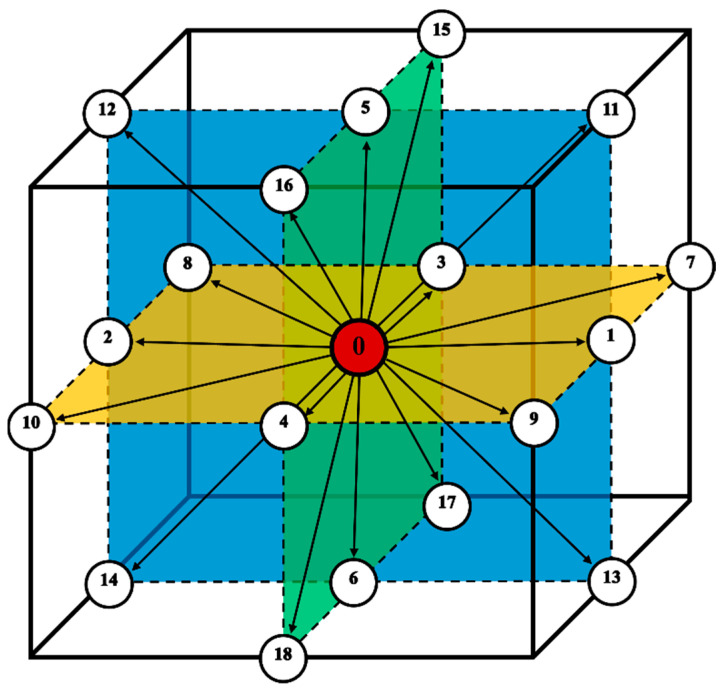
D3Q19 LBM lattice. The numbers represent particular directions, and the colors mark the perpendicular planes.

Figure 5 shows the D3Q19 velocity model [35], which is used for the simulations. The lattice velocities *e_i_* and the corresponding lattice factors for the calculation schemes are the following: *e*_(0)_ = (0,0,0), *e*_(1,2)_ = (±1,0,0), *e*_(3,4)_ = (0,±1,0), *e*_(5,6)_ = (0,0,±1), *e*_(15,16,17,18)_ = (±1,±1,0), *e*_(11,12,13,14)_ = (±1,0,±1), *e*_(7,8,9,10)_ = (0,±1,±1), and *w*_(0)_ = 1/3, *w*_(1–6)_ = 1/18, *w*_(7–18)_ = 1/36.

**Figure 6:**
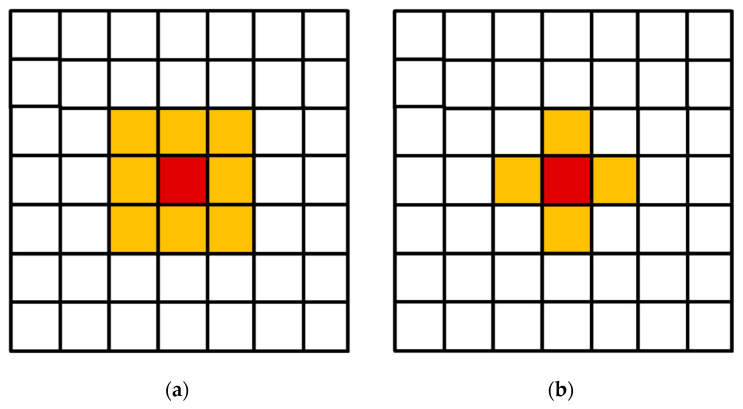
Moore (**a**) and von Neumann (**b**) neighborhoods. The cell under consideration is shown in burgundy and the cells in a given neighborhood are shown in orange.

The neighborhood is one of the most important parameters of cellular automata [36]. The simulations were performed using the three-dimensional von Neumann and Moore neighborhoods (Figure 6).

**Figure 7:**
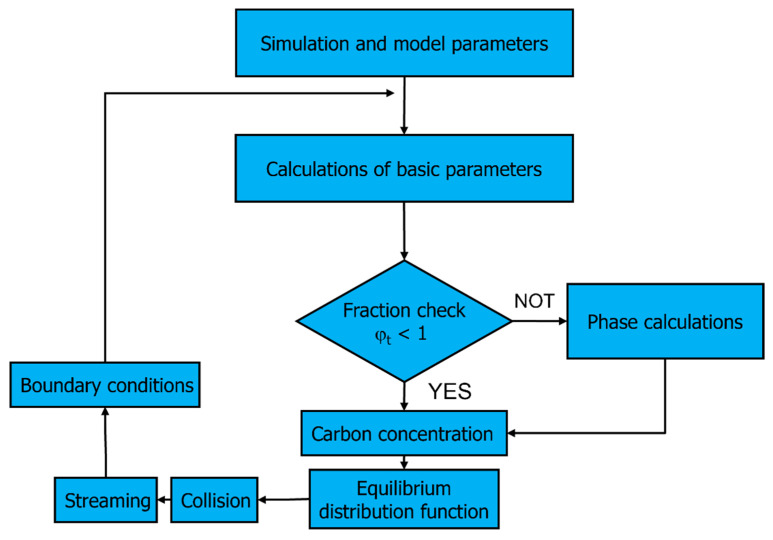
Algorithm for simulation of carbon diffusion.

Figure 7 shows an algorithm that is used to model carbon diffusion during the transformation.

The algorithm contains a cycle with an initial step. The cycle involves step nos. 2–8.

1. Initial calculation, simulation, and model parameters set:
  - Lattice sizes: *nx*, *ny,* and *nz*;
  - Nodes coordinates: *x*, *y*, *z*;
  - Lattice lengths: Δ*x*, Δ*y*, Δ*z*;
  - Time step: Δ*t*;
  - Maximal time steps: *tsteps*;
  - Austenite (*γ*) and ferrite (*α*) fractions in interface nodes: *φ_I__γ_* = 1 and *φ_I__α_* = 0;
  - Carbon concentration in nodes: *c_i_*;
  - Diffusion coefficient: *D_cf_*.
2. Calculation of basic parameters:
  - Calculation of a boundary velocity *v*:*v* = Δ*c*; Δ*c*—difference in the concentration between the interface node and the neighboring austenite nodes;
  - Calculation of changes of phase fraction in interface node: Δ*φ_I_* = *v*Δ*t*;
  - Calculation of new fraction: *φ_I__α__,t_* = *φ_I__α__,t_*_−1_ + Δ*φ_I_*.
3. State checking:
  (a) If *φ_Iα,t_* < 1, the interface state of the node remains, go to point 4;
  (b) If *φ_Iα,t_* ≥ 1, the state of the node changes from *I* (interface) to *α* (ferrite, fraction becomes *φ_I__α__,t_*′ = 1), the neighboring nodes (in the Moore neighborhood) in the state *γ* (austenite) change their state to *I* (interface), fraction for the new nodes in state *I* are calculated according to: Δ*φ_nI_* = (*φ_Iα,t_* − 1)/*numγ* = *φ_I__α__,nI_*; *numγ*—the number of nodes that changed their state from *γ* to *I*, the Δ*φ* surplus over unity: Δ*φ_oI_* = 1 − *φ_I__α_*_,*t*−1_; *φ_I__α__, oI_* = 1; *φ_I__γ__, oI_* = 0.
4. Calculation of new concentration: *c* = Σ*f_i_*;
5. Determination of the equilibrium distribution functions *f^eq^*;
6. Collision—calculation of *f^out^*;
7. Streaming—determination of *f^in^*;
8. Boundary conditions.

## 5. Simulation Results

A comprehensive multidimensional analysis was carried out on the three-dimensional model of carbon diffusion. The established fundamental linkage with the microstructure evolution model, utilizing the FCA method, enabled, among other things, the observation of transformation rates. Selected outcomes of the three-dimensional carbon diffusion modeling are showcased for specified parameter values within the model.

The first two of the modeling variants concern the modeling of carbon diffusion using the developed algorithm in a system composed of one ferrite grain located in the middle of the space. Two variants of the FCA neighborhoods were used for the simulations: von Neumann and Moore. The growth of the grain and changes in carbon concentration were modeled. The simulations were performed utilizing the D3Q19 LBM scheme (Figure 5). The modeling relies on the subsequent fundamental parameters: *n_x_* × *n_y_* × *n_z_* = 128 × 128 × 128, Δ*t* = 1, *τ* = 1, Δ*x* = 1, Δ*y* = 1, Δ*z* = 1. Boundary conditions using the bounce-back method were implemented at the edges. The initial concentration for all nodes in the calculation grid was set to 0.6. Concentration in ferrite (*α*)—0.1, austenite (*γ*)—0.9, and in interface (*c_iface_*)—0.5. The computations were executed utilizing the CUDA parallel programming architecture on graphics cards, namely, NVIDIA GeForce GTX 1060 and NVIDIA GeForce GTX 2080Ti (NVIDIA, Santa Clara, CA, USA).

**Figure 8:**
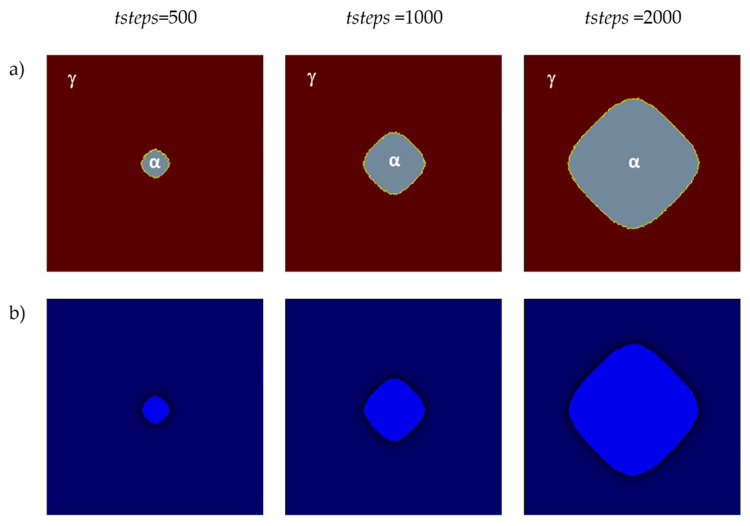
FCA states (**a**) and carbon concentration distributions (**b**) at various growth stages of the new phase utilizing the von Neumann neighborhood.

The first variant with the von Neumann neighborhood is presented in Figure 8. It shows, on the selected planes (*x*= *n_x_*/2, *y* = 1, 2, …, *n_y_*, *z* = 1, 2, …, *n_z_*) of the 3D model space, the growth of a ferrite grain (Figure 8a) and the carbon concentration distributions (Figure 8b). Different blue intensities represent different concentration values, from smaller values in light blue to larger values in dark blue.

**Figure 9:**
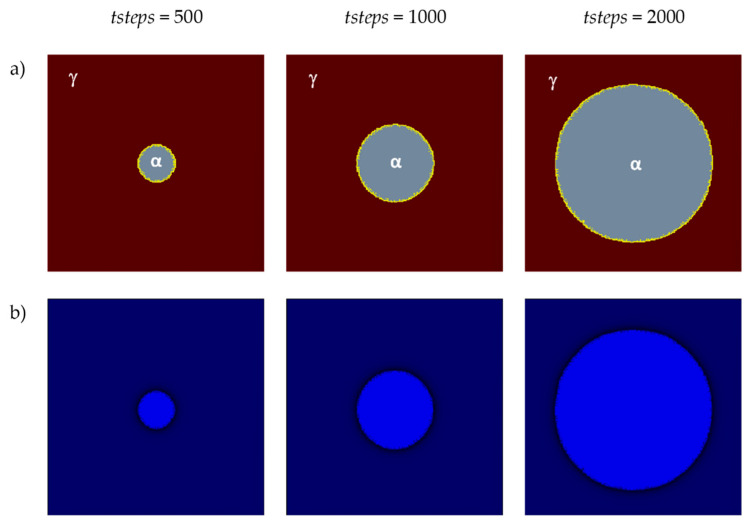
FCA states (**a**) and carbon concentration distributions (**b**) at various growth stages of the new phase utilizing the Moore neighborhood.

Figure 9a shows the second variant with the Moore neighborhood. It presents the grain growth for the identical selected plane as the variant in the von Neumann neighborhood. Distributions of carbon concentrations are presented in Figure 9b. The grain growth rate is directly related to the carbon diffusion process, and one of the main driving forces of the growth of the new phase is then the difference in carbon concentrations between the phases.

**Figure 10:**
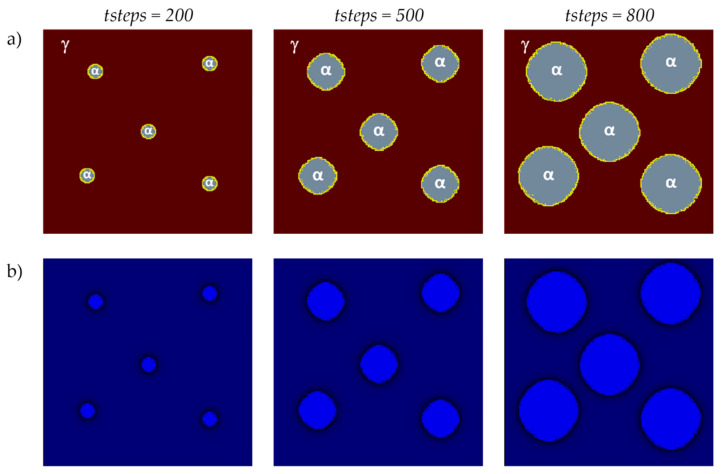
FCA states (**a**) and carbon concentration distributions (**b**) at various growth stages of the new phase grains utilizing the Moore neighborhood.

In the next presented modeling results, the microstructure evolution with five randomly located grains was analyzed. The research was carried out for another selected plane (*x* = 1, 2, …, *n_x_*, *y* = *n_y_*/2, *z* = 1, 2, …, *n_z_*) in the 3D modeled space. The basic parameters used for the modeling were as follows: a number of nodes—*n_x_* × *n_y_* × *n_z_* = 128 × 128 × 128, Δ*t* = 1, Δ*x* = 1, Δ*y* = 1, Δ*z* = 1, the bounce-back method for boundary conditions, and the Moore neighborhood. The initial concentration for all nodes in the calculation grid was set to 0.6. Concentration in ferrite—0.1, austenite—0.9, and in interface—0.5. Figure 10 shows the grains’ growth and the distribution of the carbon concentration in different stages.

**Figure 11:**
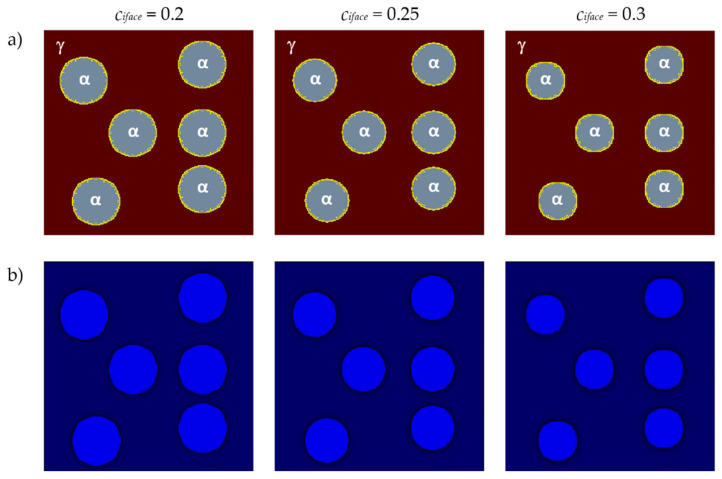
FCA states (**a**) and carbon concentration distributions (**b**) for different values of *c_iface_*.

The influence of the initial carbon concentration in interface nodes *c_iface_* on grain growth and carbon concentration distribution is shown in Figure 11. The size and shape of the grains and the carbon concentration distributions are shown for the same time step *tstep* = 400. Identical values as in the previous variant were used for the foundational parameters, and the same plane was taken into account. The growth of six randomly distributed grains of the new phase was considered.

The initial carbon concentration in interface nodes directly affects the transformation rate. The difference in concentration between the interface nodes and the neighboring austenite nodes affects the calculation of the boundary velocity. The determination of the volume of the transformed material in the interface node is directly related to the boundary velocity and affects the changes in the new phase fraction. Ferrite grains grow faster under the condition of a lower *c_iface_*.

**Figure 12:**
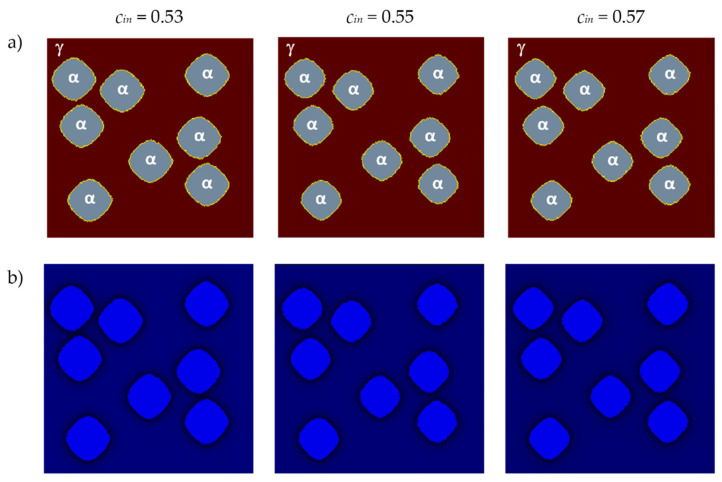
FCA states (**a**) and carbon concentration distributions (**b**) for different values of *c_in_*.

The influence of the initial carbon content *c_in_* on grain growth and carbon concentration distribution is shown in Figure 12. The size and shape of the grains and the carbon concentration distributions are shown for the same time step *tstep* = 700. The base parameters retained the same values as in the previous variant, and the analysis was conducted on the same plane. The calculations were realized with the use of the von Neumann neighborhood.

Initial carbon concentration directly affects the transformation rate. Ferrite grains grow faster under the condition of lower initial carbon content in austenite *c_in_*.

## 6. Summary

Phase transformations, especially diffusion phase transformations in carbon steels, play a crucial role in forming material properties. Numerical modeling remains pivotal in this domain, and newly developed models employ diverse approaches and methods to simulate microstructure formation after transformation. This paper presents the developed 3D carbon diffusion model for the simulation of carbon diffusion during diffusional phase transformations, which is based on the LBM. A detailed presentation of the developed carbon diffusion calculation algorithm is provided. Examples of the simulation results of carbon diffusion and the growth of the new phase in the selected planes of the 3D modeling space for different simulation conditions are also presented. The simulations used the D3Q19 LBM scheme and the von Neumann and Moore neighborhoods. The developed carbon diffusion model incorporates CUDA parallel computing on GPUs. A fundamental connection was established between the evolved carbon diffusion model and the microstructure evolution model. The developed carbon diffusion model at the next stage will also be connected to the developed 3D heat flow model during diffusional phase transformations. This will be important in the achievement of a comprehensive 3D model of carbon diffusion and heat flow during diffusional phase transformation.

## Data Availability

Data are contained within the article.
